# The predictors and surgical outcomes of different distant metastases patterns in upper tract urothelial carcinoma: A SEER-based study

**DOI:** 10.3389/fsurg.2022.1045831

**Published:** 2022-11-04

**Authors:** Xuan-han Hu, Jia Miao, Lin Qian, Da-hong Zhang, Hai-bin Wei

**Affiliations:** ^1^ The Second Clinical Medical College, Zhejiang Chinese Medical University, Hangzhou, China; ^2^ Department of Urology, Taizhou First People's Hospital, Taizhou, China; ^3^ Department of Urology, Zhejiang Provincial People's Hospital, People's Hospital of Hangzhou Medical College, Hangzhou, China

**Keywords:** upper tract urothelial carcinoma, surgery, metastatic patterns, competition risk, SEER (Surveillance epidemiology and end results) database

## Abstract

The purpose of this study was to investigate the predictors of metastatic patterns of upper tract urothelial carcinoma (UTUC) and to analyze the surgical outcomes of different metastatic patterns of UTUC. Data on patients with UTUC from 2010 to 2017 were retrieved from the Surveillance, Epidemiology, and End Results Program (SEER) database. Kaplan–Meier analysis was applied to compare the patients' survival distributions. Univariate and multivariate logistic regression was used to assess the specific predictors of site-specific metastases, while competitive risk regression was applied to estimate the predictors of cancer-specific mortality in patients with metastases. A total of 9,436 patients were enrolled from the SEER database, of which 1,255 patients had distant metastases. Lung metastasis (42.5%) was most common and patients with single distant lymph node metastasis had a better prognosis. Clinical N stage (N1, N2, N3) was the strongest predictors of the site specific metastatic sites. Renal pelvis carcinoma was more prone to develop lung metastases (*OR *= 1.67, *P *< 0.01). Resection of the primary tumor site is beneficial for the prognosis of patients with metastatic UTUC, whether local tumor resection (*HR *= 0.72, *P *< 0.01) or nephroureterectomy (*HR *= 0.64, *P *< 0.01). Patients with single distant lymph node metastasis have the greatest benefit in nephroureterectomy compared to other specific-site metastases (median survival 19 months vs. 8 months). An understanding of distant metastatic patterns and surgical outcomes in patients with UTUC is important in clinical settings and helpful in the design of personalized treatment protocols.

## Introduction

upper tract urothelial carcinoma (UTUC) is a rare and highly malignant tumor, affecting only 2 in 100,000 people (1). Although the incidence of UTUC has continued to increase, with the greater use of cross-sectional imaging, UTUC only accounts for 5%–10% of urothelial carcinoma (2,3). In this population, the incidence of metastatic UTUC is even lower, estimated to be only 12%–16% at the time of initial diagnosis (4).

Due to the rarity of metastatic UTUC, few studies have reported the predictors of metastatic patterns. Matin et al. reported that lymphatic diffusion patterns are associated with the laterality and the anatomic location of the primary tumor (5). Decker et al. indicated that age was an independent predictor of distant metastases (6). However, these studies were small-cohort investigations, and the results in these study did not describe the stratification of distant metastases at each site.

Previous studies suggested that surgical interventions such as kidney-sparing surgery (KSS) or nephroureterectomy could significantly benefit patients in UTUC (7, 8). KSS such as endoscopic ablation or segmentectomy for UTUC has become one of the important options for some patients. Chen et al. pointed that endoscopic ablation is comparable to nephroureterectomy in survival outcomes and could be used as an alternative to radical nephroureterectomy (9). However, whether KSS or nephroureterectomy can benefit in metastatic UTUC has not been reported.

This study analyzes the predictive factors of site-specific metastasis in UTUC and the surgical outcomes of this population through the SEER database. An understanding of the metastatic patterns and surgical outcomes of UTUC can provide guidance for clinicians to arrive at better decisions during the initial diagnosis.

## Methods

### Study population

The Surveillance, Epidemiology, and End Results Program (SEER) database is the largest publicly available oncology database in the United States, covering 34.6% of the U.S. population. The dataset was extracted from the SEER Research Plus Data, 18 Registries, Nov 2020 Sub (2000–2018). As variables associated with distant metastases could only be included after 2010, our cohort focused on 2010 to 2017. The inclusion criteria were as follows: (1) the primary tumor was identified by the International Classification of Diseases-O-3 (ICD-O-3) codes C64.9, C65.9. (2) the initial primary tumor was confirmed to be renal pelvis or ureter carcinoma, and (3) the histology was microscopically confirmed. Patients were excluded if distant metastasis, survival months, and vital status were unknown, or the tumor was a secondary lesion.

### Description of covariates

The variable analysis included age at diagnosis, sex, race, tumor site, tumor laterality, clinical T stage (AJCC, 7th edition, 2010), clinical N stage (AJCC, 7th edition, 2010), radiotherapy, chemotherapy, surgery, lymphodissection, tumor size, bone metastasis, liver metastasis, brain metastasis, lung metastasis, histology and pathological grades. Based on WHO 2016 grading standards, pathological grade was classified as “low level”, “high level”, and “unknown”. Surgery mainly included “none”, “local tumor resection”, “nephroureterectomy”. For tumor size, we reclassified patients into four groups as follows:"≤2 cm”, “>2 cm and ≤4 cm”, “>4 cm”, and “unknown”. The tumor laterality was categorized as “right”, “left”.

### Statistical analysis

Descriptive statistical methods were applied to summarize the demographic features and tumor characteristics of patients with and without metastases. The data were compared between groups using Student's t-test and chi-square test. Metastasis and survival distribution at diagnosis were evaluated, and the predictors related to metastasis were screened using a univariate and multivariate logistic regression risk model in the training set. Receiver operating characteristic (ROC) analysis was applied to evaluate the efficiency of the established model by area under curve (AUC) in the validation set. The Kaplan–Meier method and the log-rank test were applied to estimate the survival function among the different metastatic sites. The predictors of cancer-specific mortality (CSM) in metastatic and non-metastatic patients were identified using a competitive risk model. *P* values < 0.05 were considered significant. Statistical analyses were performed using IBM SPSS software (v25.0) and R package (v4.1.1).

## Results

### Demographic features

A total of 9,436 eligible patients with primary UTUC were screened from the SEER database from 2010 to 2017. In this series, 1,255 (13.3%) patients developed distant metastases at diagnosis. The median age at diagnosis was 72, with males accounting for 59.8% of cases and females accounting for 40.2% of cases.

Table 1 lists the differences in demographic features and tumor characteristics between patients with and without metastases, of which 6,566 patients were used as the training set and 2,870 patients were used as the validation set. There were no significant differences in sex or race between the groups. Compared to the patients without metastases, fewer patients diagnosed with distant metastases were treated with surgery and lymphodissection, while more patients were treated by radiotherapy and chemotherapy. Besides, patients with distant metastases had higher clinical T, N stages and larger tumor diameters. Lung metastasis (533/1255, 42.5%) was the most common type of metastasis in this population, while brain metastasis (32/1255, 2.5%) was the least common type of metastasis. Among all the patients with metastases, a total of 1,166 patients died, in which 1,047 deaths were cancer-specific.

**Table 1:** Patient demographics, stratified by presence of metastases at the time of diagnosis.

Characters	level	Without metastases at diagnosis	With metastases at diagnosis	*P*	Training set			Validation set		
					Without metastases at diagnosis	With metastases at diagnosis	*P*	Without metastases at diagnosis	With metastases at diagnosis	*P*
*n*		8181	1255		5700	866		2481	389	
Age (%)	≤60 years	1,050 (12.8)	193 (15.4)	0.003	737 (12.9)	141 (16.3)	0.001	313 (12.6)	52 (13.4)	0.912
	>60 and ≤70 years	2,101 (25.7)	337 (26.9)		1,467 (25.7)	235 (27.1)		634 (25.6)	102 (26.2)	
	>70 and ≤80 years	2,811 (34.4)	440 (35.1)		1,935 (33.9)	302 (34.9)		876 (35.3)	138 (35.5)	
	>80 years	2,219 (27.1)	285 (22.7)		1,561 (27.4)	188 (21.7)		658 (26.5)	97 (24.9)	
Race (%)	White	7,126 (87.1)	1,077 (85.8)	0.026	4,942 (86.7)	738 (85.2)	0.017	2,184 (88.0)	339 (87.1)	0.839
	Black	396 (4.8)	83 (6.6)		290 (5.1)	64 (7.4)		106 (4.3)	19 (4.9)	
	Other	659 (8.1)	95 (7.6)		468 (8.2)	64 (7.4)		191 (7.7)	31 (8.0)	
Sex (%)	Female	3,291 (40.2)	501 (39.9)	0.861	2,312 (40.6)	355 (41.0)	0.838	979 (39.5)	146 (37.5)	0.504
	Male	4,890 (59.8)	754 (60.1)		3,388 (59.4)	511 (59.0)		1,502 (60.5)	243 (62.5)	
Grade (%)	Low level	1,158 (14.2)	44 (3.5)	<0.001	808 (14.2)	32 (3.7)	<0.001	350 (14.1)	12 (3.1)	<0.001
	High level	6,545 (80.0)	748 (59.6)		4,553 (79.9)	512 (59.1)		1,992 (80.3)	236 (60.7)	
	Unknown	478 (5.8)	463 (36.9)		339 (5.9)	322 (37.2)		139 (5.6)	141 (36.2)	
Site (%)	Ureter	3,156 (38.6)	345 (27.5)	<0.001	2,174 (38.1)	244 (28.2)	<0.001	982 (39.6)	101 (26.0)	<0.001
	Renal pelvis	5,025 (61.4)	910 (72.5)		3,526 (61.9)	622 (71.8)		1,499 (60.4)	288 (74.0)	
Laterality (%)	Right	4,097 (50.1)	612 (48.8)	0.403	2,856 (50.1)	412 (47.6)	0.177	1,241 (50.0)	200 (51.4)	0.648
	Left	4,084 (49.9)	643 (51.2)		2,844 (49.9)	454 (52.4)		1,240 (50.0)	189 (48.6)	
Size (%)	≤2cm	1,469 (18.0)	48 (3.8)	<0.001	1,004 (17.6)	31 (3.6)	<0.001	465 (18.7)	17 (4.4)	<0.001
	>2cm and ≤4m	2,693 (32.9)	180 (14.3)		1,871 (32.8)	121 (14.0)		822 (33.1)	59 (15.2)	
	>4cm	2,583 (31.6)	537 (42.8)		1,810 (31.8)	382 (44.1)		773 (31.2)	155 (39.8)	
	Unknown	1,436 (17.6)	490 (39.0)		1,015 (17.8)	332 (38.3)		421 (17.0)	158 (40.6)	
T (%)	T1	2,766 (33.8)	178 (14.2)	<0.001	1,943 (34.1)	124 (14.3)	<0.001	823 (33.2)	54 (13.9)	<0.001
	T2	1,343 (16.4)	51 (4.1)		936 (16.4)	33 (3.8)		407 (16.4)	18 (4.6)	
	T3	3,022 (36.9)	315 (25.1)		2,089 (36.6)	222 (25.6)		933 (37.6)	93 (23.9)	
	T4	585 (7.2)	302 (24.1)		409 (7.2)	207 (23.9)		176 (7.1)	95 (24.4)	
	TX	465 (5.7)	409 (32.6)		323 (5.7)	280 (32.3)		142 (5.7)	129 (33.2)	
N (%)	N0	6,903 (84.4)	388 (30.9)	<0.001	4,813 (84.4)	273 (31.5)	<0.001	2,090 (84.2)	115 (29.6)	<0.001
	N1	473 (5.8)	290 (23.1)		332 (5.8)	194 (22.4)		141 (5.7)	96 (24.7)	
	N2	480 (5.9)	367 (29.2)		324 (5.7)	253 (29.2)		156 (6.3)	114 (29.3)	
	N3	29 (0.4)	20 (1.6)		19 (0.3)	15 (1.7)		10 (0.4)	5 (1.3)	
	NX	296 (3.6)	190 (15.1)		212 (3.7)	131 (15.1)		84 (3.4)	59 (15.2)	
Marriage (%)	Unmarried	2,988 (36.5)	498 (39.7)	0.043	2,108 (37.0)	349 (40.3)	0.031	880 (35.5)	149 (38.3)	0.486
	Married	4,757 (58.1)	704 (56.1)		3,274 (57.4)	484 (55.9)		1,483 (59.8)	220 (56.6)	
	Unknown	436 (5.3)	53 (4.2)		318 (5.6)	33 (3.8)		118 (4.8)	20 (5.1)	
Surgery (%)	None	868 (10.6)	832 (66.3)	<0.001	609 (10.7)	561 (64.8)	<0.001	259 (10.4)	271 (69.7)	<0.001
	Local tumor resection	1,336 (16.3)	56 (4.5)		926 (16.2)	43 (5.0)		410 (16.5)	13 (3.3)	
	Nephroureterectomy	5,977 (73.1)	367 (29.2)		4,165 (73.1)	262 (30.3)		1,812 (73.0)	105 (27.0)	
Radiotherapy (%)	No	7,841 (95.8)	1,028 (81.9)	<0.001	5,480 (96.1)	722 (83.4)	<0.001	2,361 (95.2)	306 (78.7)	<0.001
	Yes	340 (4.2)	227 (18.1)		220 (3.9)	144 (16.6)		120 (4.8)	83 (21.3)	
Chemotherapy (%)	No	6,356 (77.7)	601 (47.9)	<0.001	4,442 (77.9)	405 (46.8)	<0.001	1,914 (77.1)	196 (50.4)	<0.001
	Yes	1,825 (22.3)	654 (52.1)		1,258 (22.1)	461 (53.2)		567 (22.9)	193 (49.6)	
Lymphodissection (%)	No	5,852 (71.5)	1,011 (80.6)	<0.001	4,085 (71.7)	690 (79.7)	<0.001	1,767 (71.2)	321 (82.5)	<0.001
	Yes	2,329 (28.5)	244 (19.4)		1,615 (28.3)	176 (20.3)		714 (28.8)	68 (17.5)	
Bone metastases (%)	No	8,181 (100.0)	798 (63.6)	<0.001	5,700 (100.0)	560 (64.7)	<0.001	2,481 (100.0)	238 (61.2)	<0.001
	Yes	0 (0.0)	457 (36.4)		0 (0.0)	306 (35.3)		0 (0.0)	151 (38.8)	
Lung metastases (%)	No	8,181 (100.0)	722 (57.5)	<0.001	5,700 (100.0)	494 (57.0)	<0.001	2,481 (100.0)	228 (58.6)	<0.001
	Yes	0 (0.0)	533 (42.5)		0 (0.0)	372 (43.0)		0 (0.0)	161 (41.4)	
Liver metastases (%)	No	8,181 (100.0)	873 (69.6)	<0.001	5,700 (100.0)	614 (70.9)	<0.001	2,481 (100.0)	259 (66.6)	<0.001
	Yes	0 (0.0)	382 (30.4)		0 (0.0)	252 (29.1)		0 (0.0)	130 (33.4)	
Brain metastases (%)	No	8,181 (100.0)	1,223 (97.5)	<0.001	5,700 (100.0)	843 (97.3)	<0.001	2,481 (100.0)	380 (97.7)	<0.001
	Yes	0 (0.0)	32 (2.5)		0 (0.0)	23 (2.7)		0 (0.0)	9 (2.3)	
Distant lymph node metastases (%)	No	8,181 (100.0)	1,064 (84.8)	<0.001	5,700 (100.0)	730 (84.3)	<0.001	2,481 (100.0)	334 (85.9)	<0.001
	Yes	0 (0.0)	191 (15.2)		0 (0.0)	136 (15.7)		0 (0.0)	55 (14.1)	

### Distribution of metastasis patterns and surgical outcomes

**Figure 1:**
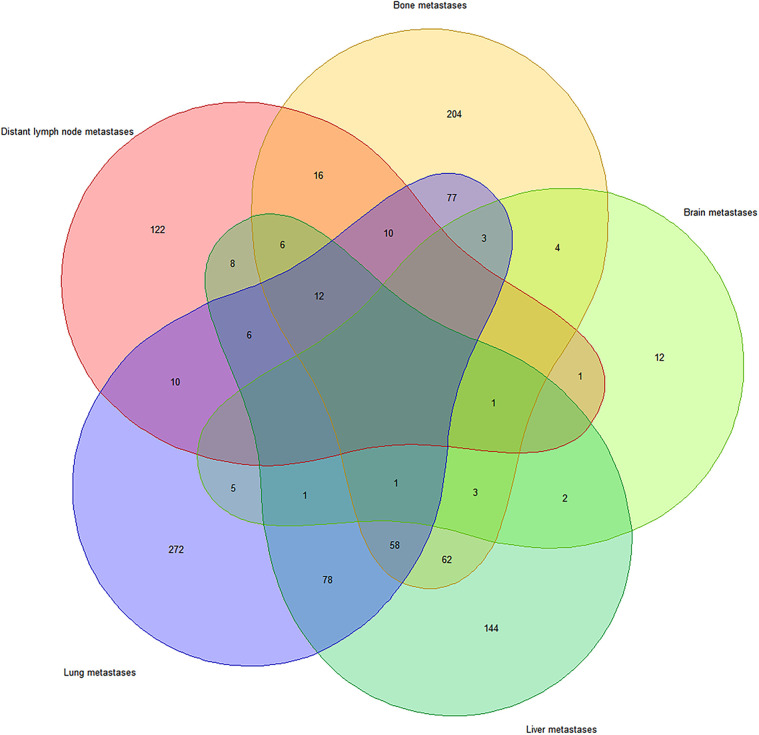
Venn diagram of the distribution of distant metastatic sites.

The Venn diagram (Figure 1) shows the distribution of distant metastases due to UTUC. Among 1,255 metastatic patients, 1,118 patients developed bone, liver, lung, brain and distant lymph node metastases. Single-site specific metastasis accounted for 60.1% (754/1255) of all cases. The lung metastases (272/754, 36.1%) was the most common region of single-site metastasis, followed by bone metastasis (204/754, 27.0%), liver metastasis (144/754, 19.1%), distant lymph node metastasis (122/754, 16.2%), and brain metastasis (12/754, 1.6%). Multiple specific metastases accounted for 29.0% (364/1255) of all cases, and simultaneous metastases of the lung and the liver (78/364, 21.4%) were the most common.

Metastatic patterns and surgical outcomes of metastatic UTUC are shown in Table 2. Among cases of single-site metastasis without surgery, patients with distant lymph node metastases had the best 1-, 3-, and 5-year overall survival (OS) and the longest median survival (8 months). Patients with brain metastases had the shortest 1-, 3-, and 5-year OS, while patients with liver metastases or brain metastases had the shortest median survival (3 months). For patients with single metastases who had undergone nephroureterectomy, the 1-year, 3-year, and 5-year OS and median survival time were significantly improved at site-specific metastasis compared with those without surgery. Among multiple metastasis, patients undergoing nephroureterectomy had a better OS and median survival time (8 months vs. 4 months).

**Table 2:** Effect of surgery on 1, 3, and 5-year OS in patients with single specific metastasis and multiple metastases.

Characters	Number (%)	1-year overall survival rate (%)	3-year overall survival rate (%)	5-year overall survival rate (%)	Median survival time (months)
Single metastasis without surgery					
Bone metastasis	137 (27.9)	21.1	2.9	0	4
Liver metastasis	95 (19.3)	10.5	4.5	1.1	3
Lung metastasis	176 (35.8)	26.1	4.2	1.0	6
Brain metastasis	9 (1.8)	0.0	0.0	0.0	3
Distant lymph node metastases	56 (15.1)	32.4	6.7	1.3	8
Single metastasis with nephroureterectomy					
Bone metastasis	59 (26.2)	27.1	11.8	1.6	8
Liver metastasis	42 (18.6)	13.0	7.3	0	5
Lung metastasis	81 (36.0)	32.4	13.5	4.1	8
Brain metastasis	2 (0.01)	33.3	0.0	0.0	6
Distant lymph node metastases	41 (18.2)	77.6	26.8	5.6	19
Multiple metastases without surgery	281 (76.1)	16.7	1.0	0.7	4
Multiple metastases with nephroureterectomy	71 (20.0)	24.2	1.3	0	8

### Predictors of metastatic disease at diagnosis

**Figure 2:**
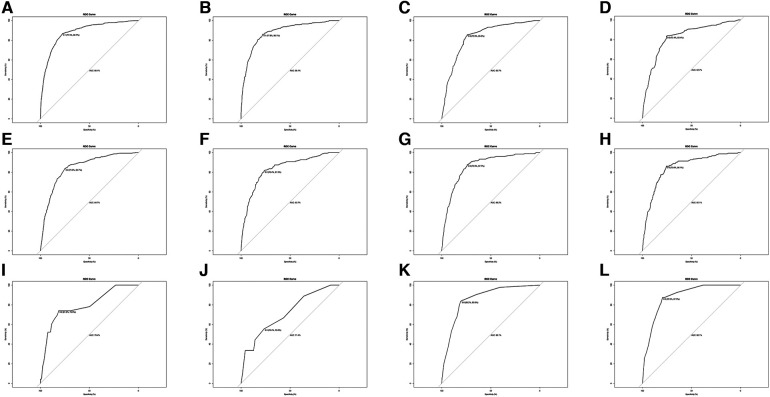
ROC analysis for evaluating the discrimination of logistic model. (**A**) ROC in the training set for metastases at all sites. (**B**) ROC in the validation set. for metastases at all sites. (**C**) ROC in the training set for lung metastases. (**C**) ROC in the validation set for lung metastases. (**E**) ROC in the training set for bone metastases (**F**) ROC in the validation set for bone metastases. (**G**) ROC in the training set for liver metastases. (**H**) ROC in the validation set for liver metastases. (**I**) ROC in the training set for brain metastases. (**J**) ROC in the validation set for brain metastases. (**K**) ROC in the training set for distant lymph node metastases. (**L**) ROC in the validation set for distant lymph node metastases.

Univariate and multivariate logistic regression was performed to analyze the predictors of metastasis at diagnosis. Table 3 summarizes the factors associated with distant metastases in the training set. Factors, such as renal pelvis tumor, grade (high level), size (>4 cm), clinical T stage (T3, T4) and clinical N stage (N1, N2, N3) were strongly associated with metastatic disease at diagnosis. Interestingly, age >80 years decreased the probability of distant metastases. Subsequently, in order to verify the validity of the model, we used the validation set to evaluate the training set model. The AUC of the training set was 0.881 (95% CI, 0.775–0.867), consistent with that in the validation set (0.881, 95%CI, 0.778–0.861), which indicated the good efficiency of the model (Figure 2).

**Table 3:** Summary of significant predictors of distant metastases at the time of diagnosis based on univariate and multivariable logistic regression hazard analysis.

Characters	Univariate				Multivariable			
	OR	95% CI for OR		*P*	OR	95% CI for OR		*P*
		Lower	Higher			Lower	Higher	
Age								
≤60 years			reference					
>60 and ≤70 years				0.124				
>70 and ≤80 years				0.066				
>80 years	0.62	0.49	0.79	<0.001	0.60	0.45	0.81	<0.001
Sex								
Female			reference					
Male				0.810				
Race								
White			reference				reference	
Black	1.47	1.10	1.94	0.006				0.431
Other	1.12	0.92	1.36	0.526				0.864
Site								
Ureter			reference				reference	
Renal pelvis	1.57	1.34	1.84	<0.001				0.161
Grade								
Low level			reference				reference	
High level	2.83	2.00	4.16	<0.001	1.60	1.09	2.44	0.020
Size								
≤2 cm			reference				reference	
>2 cm and ≤4 cm	2.09	1.42	3.18	<0.001	1.71	1.12	2.68	0.014
>4 cm	6.83	4.78	8.13	<0.001	3.33	2.23	5.12	＜0.001
T								
T1			reference				reference	
T2	0.55	0.36	0.80	0.002				0.307
T3	1.66	1.32	2.09	<0.001	1.14	1.02	1.49	<0.001
T4	7.93	6.20	10.17	<0.001	2.79	2.07	3.76	<0.001
N								
N0			reference				reference	
N1	10.30	8.30	12.77	<0.001	5.99	4.69	7.64	<0.001
N2	13.76	11.22	16.90	<0.001	7.72	6.12	9.75	<0.001
N3	13.91	6.89	27.62	<0.001	5.86	2.72	12.42	<0.001
Laterality								
Right			reference					
Left				0.165				
Marriage								
Unmarried			reference					
Married				0.134				

Abbreviations: OR, Odds Ratio; CI, Confidence Interval.

The predictors of metastasis at each site are listed in Table 4. Most of the findings were consistent with those presented in Table 2, and certain variables were associated with site-specific metastases at diagnosis. Clinical N stage (N1, N2) were significant predictors for all metastatic sites, clinical T stage (T4) was strongly associated with bone, liver and brain metastases. Age >80 years seemed to be more prone to develop bone, liver, and brain metastases. Interestingly, renal pelvis cancer was more prone to lung metastases. Tumor size (>4 cm) was also a strong predictor of liver, bone, distant lymph node and lung metastases. Gender, marriage, and laterality were not correlated with site-specific metastases. Through the ROC curve, we also compared the AUC of the training set and the validation set in the above model, and the efficiency of each model was proved.

**Table 4:** Summary of significant predictors of specific distant metastases at the time of diagnosis based on univariate and multivariable logistic regression hazard analysis.

Metastasis site	Characters	Metastasis at diagnosis			
		OR	95% CI for HR		*P*
			Lower	Higher	
Predictors of lung metastases	Renal pelvis [Ureter (reference)]	1.67	1.32	2.14	<0.001
	Clinical T stage [T4 vs. T1 (reference)]	3.24	2.19	4.83	<0.001
	Clinical N stage [N1 vs. N0 (reference)]	4.55	3.30	6.25	<0.001
	Clinical N stage [N2 vs. N0 (reference)]	4.96	3.65	6.72	<0.001
	Clinical N stage [N3 vs. N0 (reference)]	2.30	1.13	6.23	0.017
	Size > 4 cm [≤2 cm (reference)]	3.23	1.84	6.14	<0.001
Predictors of bone metastases	Age > 80 years [≤60 years (reference)]	0.53	0.35	0.80	<0.001
	Size > 4cm [≤2cm (reference)]	1.87	1.04	3.61	0.045
	Clinical T stage [T2 vs. T1 (reference)]	0.56	0.28	0.88	0.043
	Clinical T stage [T4 vs. T1 (reference)]	2.22	1.45	3.44	<0.001
	Clinical N stage [N1 vs. N0 (reference)]	4.41	3.03	6.37	0.005
	Clinical N stage [N2 vs. N0 (reference)]	6.03	4.29	8.49	<0.001
	Clinical N stage [N3 vs. N0 (reference)]	3.71	1.05	10.14	0.004
Predictors of liver metastases	Age > 80 years [≤60 years (reference)]	0.67	0.43	0.94	<0.001
	Size > 4 cm [≤2 cm (reference)]	3.41	1.65	8.26	0.002
	Grade high level [low level (reference)]	2.05	1.01	4.93	0.044
	Clinical T stage [T4 vs. T1 (reference)]	2.63	1.66	4.23	<0.001
	Clinical N stage [N1 vs. N0 (reference)]	4.51	3.03	6.69	<0.001
	Clinical N stage [N2 vs. N0 (reference)]	5.36	3.53	7.81	<0.001
Predictors of brain metastases	Age > 80 years [≤60 years (reference)]	0.89	0.87	0.94	0.027
	Clinical N stage [N1 vs. N0 (reference)]	6.58	2.02	22.15	0.001
	Clinical N stage [N3 vs. N0 (reference)]	11.71	5.82	18.34	0.031
Predictors of distant lymph node metastases	Size > 4 cm [≤2 cm (reference)]	2.24	1.13	6.01	0.034
	Clinical N stage [N1 vs. N0 (reference)]	3.41	2.55	7.33	<0.001
	Clinical N stage [N2 vs. N0 (reference)]	5.24	3.27	8.27	<0.001
	Clinical N stage [N3 vs. N0 (reference)]	5.98	3.58	9.43	<0.001

Abbreviations: OR, Odds Ratio; CI, Confidence Interval.

### Associations between metastatic sites and survival outcomes

**Figure 3:**
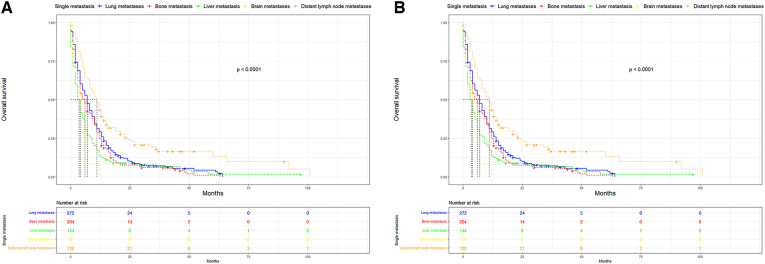
Kaplan-Meier curves of OS and CSS according to single site-specific metastasis. (**A**) OS in single site-specific metastasis (**B**) CSS in single site-specific metastasis. OS, overall survival, CSS, cancer-specific survival.

**Figure 4:**
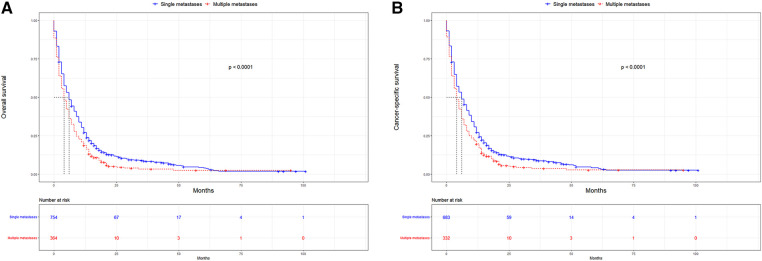
Kaplan-Meier curves for OS and CSS according to number of distant metastases. (**A**) OS in single metastasis and multiple metastases; (**B**) CSS in single metastasis and multiple metastases. OS, overall survival, CSS, cancer-specific survival.

**Figure 5:**
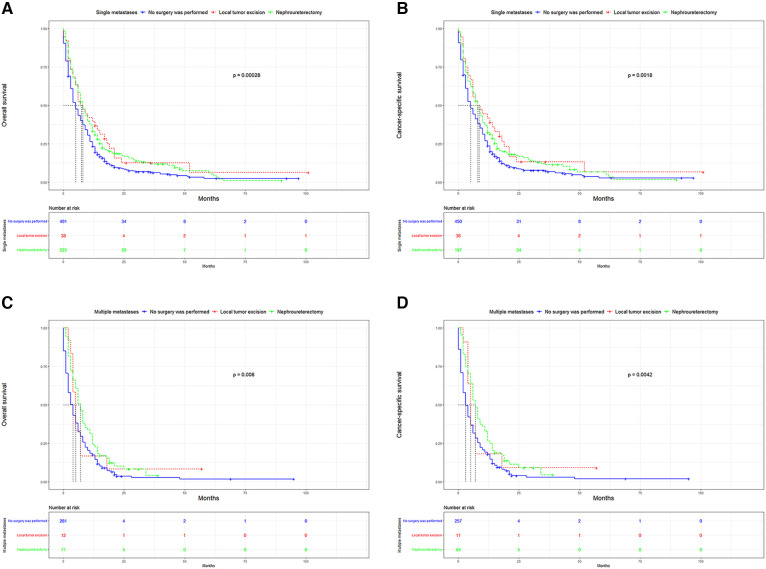
Kaplan-Meier curves for OS and CSS according to surgical methods. (**A**) OS in single metastasis; (**B**) CSS in single metastasis; (**C**) OS in multiple metastasis; (**D**) CSS in multiple metastasis. OS,overall survival; CSS, cancer-specific survival.

UTUC associates with poor prognosis, especially in patients with metastases. In the short term, single distant lymph node metastases had better OS, cancer specific survival (CSS), and median survival time compared to other single metastasis, whereas the 5-year overall survival rate for all single-site metastases is close to 0 (Figure 3). Figure 4 showed that patients with multiple metastases had a rapid decline in OS and CSS within 1 year, and the 10-year overall survival rate is 0 regardless of single or multiple metastases. Figure 5 suggested that the resection of the primary tumor, including local tumor resection and nephroureterectomy, could prolong the survival time of patients who had developed distant metastases in the short term, whether it is single or multiple metastases.

### Multivariate competitive risk analysis

Competitive risk regression analysis was performed to assess CSM. The factors affecting CSM in patients with and without metastases at diagnosis are listed in Table 5. We observed that age, tumor size, race, clinical N stage and radiotherapy were uncorrelated with CSM in patients with metastases. Chemotherapy was conducive to the survival of patients with metastases (*HR *= 0.63, *P *< 0.001), however, chemotherapy is not associated with CSM in non-metastatic UTUC (*P* > 0.05). Patients can benefit from either local excision of the primary tumor or nephroureterectomy both in metastatic UTUC and non-metastatic UTUC. Only T4 stage affected the prognosis of patients with metastases (*HR *= 1.43, *P *< 0.001). Men had better prognosis than female in metastatic UTUC (*HR *= 0.63, *P =* 0.013). Although metastases at all sites could lead to poor prognosis, distant lymph node metastases have a relatively good prognosis compared to other metastases (*HR *= 0.80, *P *< 0.001).

**Table 5:** Competing regression analysis predicting cancer-specific mortality in patients with and without metastases.

	Without Metastasis at diagnosis				Metastasis at diagnosis			
	HR	95% CI for HR		*P*	HR	95% CI for HR		*P*
		Lower	Higher			Lower	Higher	
Age								
≤60			reference				reference	
>60 and ≤70	1.28	1.09	1.49	<0.001				0.862
>70 and ≤80	1.62	1.40	1.88	<0.001				0.286
>80	2.25	1.93	2.62	<0.001				0.311
Sex								
Female							reference	
Male				0.700	0.86	0.77	0.97	0.013
Race								
White			reference				reference	
Black				0.132				0.192
Other				0.749				0.628
Site								
Ureter			reference				reference	
Renal pelvis	0.90	0.82	0.99	0.029				0.188
Grade								
Low level			reference				reference	
High level	1.74	1.49	2.03	<0.001	1.29	1.01	1.66	0.044
Size								
≤2 cm			reference				reference	
>2 cm and ≤4 cm				0.618				0.433
>4 cm	1.38	1.20	1.58	<0.001				0.461
T								
T1			reference				reference	
T2	1.40	1.22	1.61	<0.001				0.167
T3	2.18	1.94	2.44	<0.001				0.433
T4	3.67	3.09	4.36	<0.001	1.43	1.17	1.75	<0.001
N								
N0			reference				reference	
N1	2.09	1.77	2.47	<0.001				0.190
N2	2.15	1.83	2.54	<0.001				0.195
N3	2.62	1.46	4.76	v				0.611
Surgery								
None			reference				reference	
Local tumor resection	0.43	0.36	0.52	<0.001	0.72	0.59	0.89	0.003
Nephroureterectomy	0.42	0.34	0.49	<0.001	0.64	0.53	0.76	<0.001
Lymphodissection								
No			reference				reference	
Yes	0.77	0.69	0.85	<0.001				0.632
Radiotherapy								
No			reference				reference	
Yes	1.39	1.16	1.67	<0.001				0.515
Chemotherapy								
No			reference				reference	
Yes				0.167	0.63	0.55	0.71	<0.001
Liver metastases								
No							reference	
Yes					1.23	1.05	1.45	0.014
Bone metastases								
No							reference	
Yes					1.35	1.15	1.58	<0.001
Brain metastases								
No							reference	
Yes					1.50	1.04	2.70	<0.001
Lung metastases								
No							reference	
Yes					1.18	1.02	1.37	0.025
Distant lymph node metastases								
No							reference	
Yes					0.80	0.66	0.96	0.019

HR, Hazard Ratio; CI, Confidence interval.

## Discussion

Metastatic UTUC is extremely rare, and its incidence has been gradually increasing in the past three decades, with a significant increase from 0.1 to 0.4 per 100,000 individuals (10,11). In this study, 17.2% of patients presented with distant metastases, and the higher incidence may be due to the increased use of positron emission tomography/computed tomography and bone scanning in recent years. The prediction of tumor metastatic patterns is important in clinical decision making in cancers of the prostate, kidney, and testis (12–14). However, few studies have focused on UTUC (15). Thus, it is important to define the independent predictors of metastasis, which will help us to further understand the disease.

In addition to the overall metastatic rate of UUTC, we also evaluated the distribution of the different metastatic sites. Similar to a previous study, lung metastasis was the most common, followed by bone and liver metastases, with brain metastasis being relatively rare (16). Many studies have reported that UTUC associated with very poor OS and CSS (17,18). This was also the case in this study. After comparing the survival distribution with the log-rank test results, we observed that single distant lymph node metastases had the best short-term prognosis, with brain metastases being the worst. The 5-year overall survival rates for each single metastasis were close to 0, suggesting a poor prognosis for metastatic UTUC. Subsequently, we found that primary tumor resection, especially nephroureterectomy, significantly improved median survival, OS and CSS in the short-term. Patients with single distant lymph node metastasis had the most significant improvement after nephroureterectomy compared to other single site-specific metastasis. However, long term survival of patients with a single metastasis did not improve significantly after surgery. For multiple metastases, patients can still benefit from surgery. However, for most patients with multiple metastases, their survival is less than 1 year, the impact of surgical trauma and renal impairment on the patients must be considered. For patients with a single metastasis, nephroureterectomy may be recommended.

In a subsequent study, we compared the independent predictors of distant and site-specific metastases. Age was an independent predictor for both distant and site-specific metastases, while gender had no effect on both, consistent with Deuker et al. (6). In addition, we observed that clinical T stage (T4) and clinical N stage (N1, N2) were the strongest predictors of distant and site-specific metastases. Li et al. also reported a similar view, that is, stage IV was a high risk factor for metastasis (16). In patients with T4 stage tumors, there is invasion of perirenal organs or blood vessels, tumors are prone to progress to hematogenous metastasis. For patients with regional lymph node infiltration, the probability of distant lymph node metastasis was higher.

We observed that renal pelvis cancer was an independent predictor of lung metastasis. However, the underlying mechanisms are unclear and further studies are still needed. Tumor size (>4 cm) was a risk factor for liver, lung, liver and distant lymph node metastases, which is consistent with the risk stratification criteria described in the EAU guidelines (4).

In competitive risk regression analysis, tumor sites and tumor size affected CSM only in non-metastatic UTUC, but not in metastatic UTUC. Lwin et al. had a similar point, they believed the reason why ureteral cancer associated with poorer CSM was due to the high stage of ureteral cancer in non-metastatic UTUC and the unsatisfactory treatment effect (19). We conjecture that once distant metastases have occurred, tumor biological behavior has a greater impact on prognosis, while the influence of local tumor factors is relatively reduced.

Interestingly, we observed that women had poorer CSM in metastatic UTUC, which was also supported by Mohamad et al., who suggested that this may be associated with later diagnosis, higher T stage, and higher *N* stage in women (20). Other potential causes may be due to a combination of metabolic and genetic, anatomical, hormonal and environmental factors.

For metastatic UTUC, radiotherapy didn’t improve patients' outcomes. In this study, metastatic UTUC can benefit from chemotherapy, either postoperative adjuvant chemotherapy or end-stage salvage chemotherapy. This is why chemotherapy has always been the best choice for the palliative treatment of patients with metastatic UTUC (21). Nephroureterectomy delayed tumor progression and improved outcomes in metastatic and non-metastatic UTUC, whereas lymphatic dissection improved survival outcomes only in non-metastatic patients. These results suggest that at different stages of the disease, clinicians can choose different surgical procedures and chemotherapeutic combinations to achieve the best therapeutic effect (22).

Among all site-specific metastases, distant lymph node metastases had less impact on CSM, which is consistent with the results of survival analysis. We believed this was due to the fact that we could still manage distant lymph node metastases with various means such as lymph node dissection, chemotherapy and immunotherapy (23). Although brain metastases were less likely to occur, they had the greatest impact on CSM, as most patients had only a 1-year survival. This may be related to factors such as secondary intracranial hypertension and tumor hemorrhage.

Although this investigation strictly followed the study design, there were still several shortcomings. Firstly, the SEER database only provides information on lung, bone, liver, brain and distant lymph node metastases after 2010, and no study has been performed on the other types of metastasis. Secondly, in terms of surgery, radiotherapy, chemotherapy, and other variables in the data, we are not clear about the treatment sequence and whether comprehensive treatment should be performed. Since the SEER database did not provide further description of functional characteristics, the complications and functional status of the patients were not analyzed in this study.

## Conclusion

Overall, 13.3% of patients developed distant metastases at diagnosis. Patients with lung metastases were the most common. Single distant lymph node metastasis had the best oncologic survival, while those with brain metastases showed the worst prognosis among single metastases. We identified several specific factors associated with site-specific metastasis. Renal pelvis cancer was more prone to lung metastasis. Clinical *N* stage were all sites risk factors for metastases at all sites. Tumor size (>4 cm) was a strong predictor of liver, bone, distant lymph node and lung metastases. CSM was higher in women with metastatic UTUC. Resection of the primary tumor, especially nephroureterectomy, can benefit patients with distant metastases, with the most obvious improvement in survival time for single distant lymph node metastasis. Maintenance chemotherapy also improved patient outcomes. For patients with metastatic UTUC, understanding the metastatic patterns, the factors inducing metastasis, and the surgical outcomes on survival of patients with metastases at different sites is helpful for personalized interventions.

## Data Availability

The raw data supporting the conclusions of this article will be made available by the authors, without undue reservation.

## References

[1] Killock D. New standard for localized UTUC. Nat Rev Clin Oncol. (2020) 17(5):275. 10.1038/s41571-020-0354-6 32203273

[2] van Doeveren T van der Mark M van Leeuwen PJ Boormans JL Aben KKH. Rising incidence rates and unaltered survival rates for primary upper urinary tract urothelial carcinoma: a Dutch population-based study from 1993 to 2017. BJU Int. (2021) 128(3):343–51. 10.1111/bju.15389 33690922 PMC8453942

[3] Zeng S Ying Y Yu X Wang L Zhang Z Xu C. Impact of previous, simultaneous or intravesical recurrence bladder cancer on prognosis of upper tract urothelial carcinoma after nephroureterectomy: a large population-based study. Transl Androl Urol. (2021) 10(12):4365–75. 10.21037/tau-21-758 35070818 PMC8749064

[4] Rouprêt M Babjuk M Burger M Capoun O Cohen D Compérat EM European association of urology guidelines on upper urinary tract urothelial carcinoma: 2020 update. Eur Urol. (2021) 79(1):62–79. 10.1016/j.eururo.2020.05.042 32593530

[5] Matin SF Sfakianos JP Espiritu PN Coleman JA Spiess PE. Patterns of lymphatic metastases in upper tract urothelial carcinoma and proposed dissection templates. J Urol. (2015) 194(6):1567–74. 10.1016/j.juro.2015.06.077 26094807 PMC4896731

[6] Deuker M Rosiello G Stolzenbach LF Martin T Collà Ruvolo C Nocera L. Sex- and age-related differences in the distribution of metastases in patients with upper urinary tract urothelial carcinoma. J Natl Compr Canc Netw. ((2021) Feb 11) 19(5):534–40. 10.6004/jnccn.2020.7637 33571954

[7] Ng Chieng Hin J Hettiarachchilage D Gravestock P Rai B Somani BK Veeratterapillay R. Role of ureteroscopy in treatment of upper tract urothelial carcinoma. Curr Urol Rep. (2021) 22(10):49. 10.1007/s11934-021-01065-7 34622345 PMC8497313

[8] Pathak RA Hemal AK. Techniques and outcomes of robot-assisted nephro-ureterectomy for upper tract urothelial carcinoma. Eur Urol Focus. (2018) 4(5):657–61. 10.1016/j.euf.2018.08.007 30146238

[9] Chen YT Yu CC Yeh HC Lee HY Jiang YH Lee YK Endoscopic management versus radical nephroureterectomy for localized upper tract urothelial carcinoma in a high endemic region. Sci Rep. (2021) 11(1):4040. 10.1038/s41598-021-83495-4 33597574 PMC7889610

[10] Leow JJ Chong KT Chang SL Bellmunt J. Upper tract urothelial carcinoma: a different disease entity in terms of management. ESMO Open. (2017) 1(6):e000126. 10.1136/esmoopen-2016-000126 28848663 PMC5419214

[11] Zhang X Wang P Qi K Qiao Q Jiang Y. The role of surgery on primary site in metastatic upper urinary tract urothelial carcinoma and a nomogram for predicting the survival of patients with metastatic upper urinary tract urothelial carcinoma. Cancer Med. (2021) 10(22):8079–90. 10.1002/cam4.4327 34647688 PMC8607251

[12] Shou J Zhang Q Wang S Zhang D. The prognosis of different distant metastases patterns in prostate cancer: a population based retrospective study. Prostate. (2018) 78(7):491–7. 10.1002/pros.23492 29436722

[13] Chandrasekar T Klaassen Z Goldberg H Kulkarni GS Hamilton RJ Fleshner NE. Metastatic renal cell carcinoma: patterns and predictors of metastases-A contemporary population-based series. Urol Oncol. (2017) 35(11):661.e7–661.e14. 10.1016/j.urolonc.2017.06.060 28728748

[14] Xu P Wang J Abudurexiti M Jin S Wu J Shen Y Prognosis of patients with testicular carcinoma is dependent on metastatic site. Front Oncol. (2020) 10(9):1495. 10.3389/fonc.2019.01495 31998648 PMC6966605

[15] Shinagare AB Fennessy FM Ramaiya NH Jagannathan JP Taplin ME Van den Abbeele AD. Urothelial cancers of the upper urinary tract: metastatic patterns and its correlation with tumor histopathology and location. J Comput Assist Tomogr. (2011) 35(2):217–22. 10.1097/RCT.0b013e31820d7a37 21412093

[16] Li X Li S Chi Z Cui C Si L Yan X Clinicopathological characteristics, prognosis, and chemosensitivity in patients with metastatic upper tract urothelial carcinoma. Urol Oncol. (2021) 39(1):75.e1–8. 10.1016/j.urolonc.2020.06.010 32654950

[17] Tanaka N Kikuchi E Kanao K Matsumoto K Kobayashi H Miyazaki Y Patient characteristics and outcomes in metastatic upper tract urothelial carcinoma after radical nephroureterectomy: the experience of Japanese multi-institutions. BJU Int. (2013) 112(2):E28–34. 10.1111/bju.12133 23795795

[18] Seisen T Jindal T Karabon P Sood A Bellmunt J Rouprêt M Efficacy of systemic chemotherapy plus radical nephroureterectomy for metastatic upper tract urothelial carcinoma. Eur Urol. (2017) 71(5):714–8. 10.1016/j.eururo.2016.11.012 27912971

[19] Lwin AA Hsu CH Chipollini J. Urothelial carcinoma of the renal pelvis and ureter: does location make a difference? Clin Genitourin Cancer. (2020) 18(1):45–9.e1. 10.1016/j.clgc.2019.10.023 31786118

[20] Al-Ali B M Madersbacher S Zielonke N Schauer I Waldhoer T Haidinger G. Impact of gender on tumor stage and survival of upper urinary tract urothelial cancer: a population-based study. Wien Klin Wochenschr. (2017) 129(11-12):385–90. 10.1007/s00508-016-1088-4 27670858 PMC5486730

[21] Chang YH Hsiao PJ Chen GH Lin CC Chang CH Wu HC Outcomes of stage II-IV upper-tract urothelial carcinoma and adjuvant chemotherapy for locally advanced cancer. Oncol Lett. (2019) 17(1):1341–8. 10.3892/ol.2018.9672 30655904 PMC6313078

[22] Murakami Y Matsumoto K Ikeda M Hirayama T Utsunomiya T Koguchi D Impact of histologic variants on the oncological outcomes of patients with upper urinary tract cancers treated with radical surgery: a multi-institutional retrospective study. Int J Clin Oncol. (2019) 24(11):1412–8. 10.1007/s10147-019-01486-y 31197556

[23] Freifeld Y Krabbe LM Clinton TN Woldu SL Margulis V. Therapeutic strategies for upper tract urothelial carcinoma. Expert Rev Anticancer Ther. (2018) 18(8):765–74. 10.1080/14737140.2018.1481395 29848133

